# Establishing a Detection Method Based on Multiplex PCR for Identification of Sheep Meat, Goat Meat and Common Adulterant Meats

**DOI:** 10.3390/foods14223875

**Published:** 2025-11-13

**Authors:** Yanbing Yang, Kai Quan, Huiguo Yang, Yuxuan Song, Xiyun Zhang, Bo Wang, Xiaoyang Lv, Wei Sun

**Affiliations:** 1 College of Animal Science and Technology, Yangzhou University, Yangzhou 225009, China; 2 Joint International Research Laboratory of Agriculture and Agri-Product Safety of Ministry of Education, Yangzhou University, Yangzhou 225009, China; 3 College of Animal Science and Technology, Henan University of Animal Husbandry and Economics, Zhengzhou 450046, China; quankai1115@163.com; 4 Institute of Animal Husbandry, Xinjiang Academy of Animal Sciences, Urumqi 830013, China; yanghuiguo3239@163.com; 5 College of Animal Science and Technology, Northwest A&F University, Yangling 712100, China; syx98728@163.com; 6 Gansu Yuansheng Agriculture & Animal Husbandry Technology Co., Ltd., Jinchang 737200, China; ycys_zxy@163.com; 7 College of Food Science and Engineering, Yangzhou University, Yangzhou 225127, China; wb@yzu.edu.cn; 8 International Joint Research Laboratory in Universities of Jiangsu Province of China for Domestic Animal Germplasm Resources and Genetic Improvement, Yangzhou University, Yangzhou 225009, China

**Keywords:** qualitatively identify, mitochondrial complete genome sequences, species-specific primers, authenticity

## Abstract

This study aimed to establish a multiplex PCR identification system capable of rapidly detecting adulteration in sheep and goat meat, while qualitatively identifying common adulterant meats (pork, chicken, and duck). Species-specific primers targeting mitochondrial DNA sequences were designed after screening for gene fragments with intraspecies conservation and interspecies specificity across five target species. The multiplex PCR conditions and system were systematically optimized and evaluated for specificity, reproducibility, sensitivity, and practical applicability using simulated mixed samples and heat-treated products. The results demonstrated that the system could successfully identify sheep meat, goat meat, and adulterant meat components in randomly combined target meat template DNAs with excellent reproducibility. The system maintained a high sensitivity, detecting target species even at low DNA template concentrations and in samples with low adulteration ratios. Moreover, target meat components remained detectable in heat-treated products, confirming the system’s robustness under realistic market conditions. This multiplex PCR identification system demonstrates strong specificity, good reproducibility, high sensitivity, and broad applicability. It provides an important tool for effectively monitoring sheep and goat meat adulteration and offers crucial technical support for ensuring the authenticity of sheep and goat meat.

## 1. Introduction

Sheep and goat meat has long been valued for its delicious taste, high nutritional value, and therapeutic properties in Chinese culinary culture. Global meat production reached 379 million tons in 2024, representing a 1.7% increase from the previous year according to the Food and Agriculture Organization of the United Nations (FAO) [1]. The global meat market has undergone a significant structural transformation. Poultry meat is currently estimated to account for 35–41% of global meat production, surpassing traditional meat types [2]. International meat trade reached 42.5 million tons in 2024, growing by 4.7%, reflecting the increasingly interconnected nature of global meat markets [3]. In recent years, the adulteration of sheep meat and goat meat has become increasingly prominent due to two factors: the growing popularity of sheep and goat meat (such as in barbecue, hot pot) and the penetration of foreign culinary cultures, along with a high price driven by a supply–demand imbalance, import restrictions, and epidemics. The FAO Meat Price Index reached a historic high of 127.8 points in August 2025, reflecting a tight global meat supply [4]. This price volatility, combined with significant regional price disparities (particularly in Asia where meat consumption is rapidly growing), has created strong economic incentives for meat adulteration [5]. Unscrupulous merchants have added cheaper meats to sheep and goat meat products for profit, thereby disrupting market order and compromising food safety [6,7].

The scale of meat adulteration has become a significant concern in international food safety monitoring. According to the FAO statistical databases covering 245 countries and regions, the concentration of meat production and trade with the top three exporters (Brazil, the United States, and the European Union) accounts for over half of global meat exports. This concentration has created complex supply chains that increase vulnerability to fraud [8]. In developing regions, particularly in Africa where per capita meat consumption remains below 20 kg annually, the challenge of ensuring meat authenticity is compounded by limited regulatory infrastructure. The Asia-Pacific region, which has experienced the most dramatic increase in meat consumption with China’s per capita consumption recovering to pre-African Swine Fever levels, represents a particularly critical market for authentication technologies [9].

Traditional authentication methods such as sensory evaluation, chemical detection, and immunological approaches face significant limitations including poor specificity, subjective identification, a restricted application range, and complex operational requirements for processed food or mixed samples [10]. Beyond traditional methods, spectroscopic and imaging techniques coupled with advanced data analysis have emerged as promising alternatives for rapid, non-destructive authentication [11,12]. The limitations of traditional methods and the emergence of new technologies have driven the development of molecular biology techniques for food adulteration detection [13,14].

PCR-based methodologies have emerged as a leading approach for meat species identification, offering an excellent performance in terms of specificity, reproducibility, sensitivity, and applicability. Various PCR techniques have been incorporated into regulatory standards, including conventional PCR, fluorescence quantitative PCR, multiplex PCR, and digital PCR [15,16,17]. Advanced multiplexing approaches enable the simultaneous detection of multiple meat species, providing cost-effective alternatives to single-target detection systems [18,19].

However, these methods face challenges with highly processed foods due to DNA degradation during thermal processing, pH modification, and mechanical treatment. Recent innovations have focused on enhancing sensitivity while maintaining specificity, with semi-nested PCR representing a significant advancement for processed products where DNA integrity is compromised [20]. This technique utilizes a two-stage amplification with overlapping primers to achieve a substantially higher sensitivity than conventional PCR while maintaining necessary specificity. The selection of appropriate DNA targets significantly influences detection sensitivity, with mitochondrial targets offering particular advantages due to their abundance and distinctive sequence variations between species [21].

This study aims to establish a multiplex PCR amplification system for the simultaneous identification of sheep meat, goat meat, pork, chicken, and duck meat. These five species were selected as representative targets due to their prevalence and economic significance in the Chinese meat market. National consumption and price data indicate that they constitute the majority of red and white meat traded domestically and are the most frequently involved in adulteration incidents. In particular, pork, duck, and chicken are lower-cost meats often substituted for sheep or goat meat. Other potential adulterants, such as beef, rabbit, or horse, were excluded because of their limited market presence and smaller price differentials relative to sheep and goat meat in most regions of China. Through the optimization of experimental conditions, and the evaluation of specificity, reproducibility, sensitivity, and applicability using simulated adulteration experiments, this study assesses the practical performance of the system to safeguard the authenticity and food safety of sheep and goat meat products.

## 2. Materials and Methods

### 2.1. Meat Sample Sources

Six samples of goat meat and sheep meat were obtained from laboratory sampling (2023, Haimen Breeding Sheep Farm, 2025, Gansu Yuansheng Agriculture & Animal Husbandry Technology Co., Ltd., Jinchang, China), which were taken directly from the source of sheep and goat meat supplies. The remaining meat samples were randomly purchased (six samples per species) from markets near Yangzhou University (16 December 2024, Liqun Times Square, 2nd Floor Supermarket; 23 February 2025, Impression Mall, 2nd Floor Supermarket, Yangzhou, China). All market samples were obtained from licensed suppliers with clear species labeling. Thus, with five species and 6 samples per species, a total of 30 samples were studied.

### 2.2. DNA Extraction

Next, 100 mg of each meat sample was taken and the meat samples were frozen using liquid nitrogen and then ground to a powder using an autoclaved mortar and pestle (Hebei Huaou Glass Co., Ltd., Cangzhou, China). DNA was extracted using the TIANamp Genomic DNA Kit with colum type(Tiangen Biotech (Beijing) Co., Ltd., Beijing, China) according to the manufacturer’s instructions. To avoid DNA cross-contamination, measures such as partitioning operations, dedicated consumables, and equipment cleaning were applied between each extraction. The extracted DNA was quantified using NanoDrop™ One/OneC 2000/8000 Micro-Volume UV-Vis (Thermo Fisher Scientific, Waltham, MA, USA), with DNA purity ranging between 1.7 and 2.0. The DNA was then diluted to 20 mg/mL and stored at −20 °C until required.

### 2.3. Establishment of Multiplex PCR Detection Method

#### 2.3.1. Selection of Specific Primers

Mitochondrial complete genome sequences of sheep (NC_001941.1), goat (NC_005044.2), chicken (NC_053523.1), pig (NC_000845.1), and duck (NC009684.1) were downloaded from the NCBI (National Center for Biotechnology Information) GenBank database [22]. ClustalW was used to screen gene fragments with intraspecies conservatism and interspecies specificity [23]. The criteria for including specific DNA sequences were as follows: comprehensive and accurate information available in the NCBI for different species; no differences in base length between different strains of the same species; sufficient differences in base correspondence and length between different species; and a DNA sequence length under 700 bp to facilitate primer design. Five pairs of specific primers were preliminarily selected by evaluating primer self-structure (GC content, length, hairpin, dimers, false priming, and cross dimer), and the interactions between primers (dimers), annealing temperatures (differences between upstream and downstream primers and between different species), and product length (≤700 bp) [24]. Blastn (NCBI) and Autodimerv1 were used to ensure that primers would not cause non-specific amplification or cross-reactions during experiments [25]. Information on the primers and their amplification fragment lengths is shown in Table 1. All primers were synthesized by Beijing TsingKe Biological Technology Co., Ltd. (Beijing, China).

#### 2.3.2. Optimization Testing of Multiplex PCR Amplification Annealing Temperature

Using the BIO-RAD gradient PCR thermocycler (Bio-Rad Laboratories, Hercules, CA, USA), multiplex PCRs were performed by adjusting the annealing temperature and cycle number while keeping the other conditions constant to ensure clear and bright amplification bands without tailing or diffusion that are positioned at the target location and appear to be singular. The multiplex PCR amplification conditions were as follows: 94 °C, 3 min; 94 °C, 30 s; 56–60 °C, 30 s; 72 °C, 1 min; 30 cycles; 72 °C, 5 min. The blank control group was amplified with ddH_2_O.

#### 2.3.3. Agarose Gel Electrophoresis

Next, 2% agarose gel (Beijing TsingKe Biological Technology Co., Ltd., Beijing, China) containing green nucleic acid dye (Beijing Solarbio Science & Technology Co., Ltd., Beijing, China) was prepared and placed in a DYY-6C electrophoresis apparatus (Beijing Liuyi Biotechnology Co., Ltd., Beijing, China). Each PCR-amplified sample and DNA ladder (Beijing Tsingke Biotechnology Co., Ltd., Beijing, China) was loaded at 7 μL, with the voltage at 120 V and current at 150 mA for approximately 30 min. After electrophoresis, the gel was observed under Bio-Rad ChemiDoc/CellDoc gel imaging system (Bio-Rad Laboratories, Hercules, CA, USA).

### 2.4. Specificity Experiment

Extracted DNA was subjected to single PCR and multiplex PCR amplification detection. The single PCR amplification system consisted of 12.5 μL 2 × TSINGKE Master Mix (Beijing TsingKe Biological Technology Co., Ltd., Beijing, China), 0.5 μL upstream primer and 0.5 μL downstream primer (5 types of primers mixed through oscillation ), 10.5 μL ddH_2_O, and 1 μL template DNA (nmol), with a total volume of 25 μL.

The multiplex PCR amplification system consisted of 12.5 μL 2 × TSINGKE Master Mix (Beijing TsingKe Biological Technology Co., Ltd., Beijing, China), 1 μL upstream and downstream primers (a mix of five types of primers in equal proportion), 10.5 μL ddH_2_O, and 1 μL template DNA (different species randomly mixed together in equal proportion), with a total volume of 25 μL.

The PCR amplification parameters were as follows: 94 °C, 3 min; 94 °C, 30 s; 56.8 °C, 30 s; 72 °C, 1 min; 30 cycles; 72 °C, 5 min.

Single PCR and multiplex PCR amplification were performed under the same reaction conditions and detected by gel electrophoresis. Specificity was evaluated based on the following: (1) the presence of expected target bands at correct positions; (2) the absence of non-specific amplification; (3) no cross-reactivity with non-target species; (4) no amplification in blank controls.

### 2.5. Reproducibility Experiment

To evaluate the reproducibility of the multiplex PCR detection system, DNA samples from five target species (6 biological replicates from each species) were diluted to 20 ng/μL and mixed using an SI Vortex Genie2 vortex mixer (Scientific Industries, Inc., Bohemia, NY, USA) for 30 s. The primer concentration, PCR program, system conditions, and so on were kept constant (PCR amplification parameters and reaction system are detailed in Section 2.4). The DNA mixture was amplified three times by the same operator to assess the consistency of the multiplex PCR system. Amplification products were analyzed by 2% agarose gel (Beijing TsingKe Biological Technology Co., Ltd., Beijing, China) electrophoresis to ensure experimental reproducibility based on the electrophoresis results.

### 2.6. Sensitivity Experiment

To obtain DNA template concentration gradients (20 ng/μL, 10 ng/μL, 5 ng/μL, 2.5 ng/μL, 1.25 ng/μL, and 0.625 ng/μL), one animal’s DNA was diluted with ddH2O in a 1:1 series, maintaining the concentrations of DNA from other animals. Then, this was mixed with the remaining four animal DNA templates to obtain target DNA template concentration gradients (4 ng/μL, 2 ng/μL, 1 ng/μL, 0.5 ng/μL, 0.25 ng/μL, and 0.125 ng/μL) for multiplex PCR amplification (see Section 2.4. for parameters). The PCR amplification system comprises the following: 12.5 μL 2 × TSINGKE Master Mix (Beijing TsingKe Biological Technology Co., Ltd., Beijing, China), 1 μL upstream and downstream primers (mixture with equal volumes of the five primers), 10.5 μL ddH_2_O, and 0.2 μL diluted template DNA with the remaining 0.8 μL undiluted (0.2 μL per species), for a total volume of 25 μL. The detection process was conducted via 2% gel (Beijing TsingKe Biological Technology Co., Ltd., Beijing, China) electrophoresis, which was utilized to ascertain the minimum DNA concentration that could be detected by the multiplex PCR system.

### 2.7. Analog Simulation Experiment

#### 2.7.1. Simulation of Duck Meat Adulteration in Sheep and Goat Meat

In cases of adulteration involving sheep and goat meat, adulterant meat typically enters the market as inexpensive duck meat, substituting for higher-priced meat (sheep and goat) [6]. This experiment involved the simulation of actual cases, with the most common scenario being that of duck meat mixed with sheep and goat meat. Meat samples were sliced thinly and ground into a powder using a mortar (Hebei Huaou Glass Co., Ltd., Cangzhou, China), with sheep meat and goat meat being ground separately. Duck meat proportions in mixed meat samples were controlled at 50%, 25%, 10%, and 5% using Mettler PL203 Electronic Analytical Balance (Mettler-Toledo International Inc., Columbus, OH, USA). Each simulated mixed sample had a total mass of approximately 220 mg. Following a thorough mixing process, the DNA was extracted, amplified using the multiplex PCR system (refer to Section 2.4 for PCR amplification system and parameters), and detected by 2% agarose gel (Beijing TsingKe Biological Technology Co., Ltd., Beijing, China) electrophoresis.

#### 2.7.2. Heat-Treated Meat Product Adulteration Experiment

Five meat products of the target species, heat-treated at 100 °C (10 min) using microwave (Guangzhou Galanz Group Co.,Ltd., Shunde, China) processing, were subjected to DNA extraction [26]. The extracted DNA was diluted to a concentration of 20 ng/μL. The operation detailed in Section 2.4 was repeated for the purpose of PCR amplification, and the results were detected using 2% gel (Beijing TsingKe Biological Technology Co., Ltd., Beijing, China) electrophoresis. This process was employed in order to verify the suitability of the system to the complex environment of market adulteration.

## 3. Results

### 3.1. Optimization Ressult of Multiplex PCR Annealing Temperature

**Figure 1:**
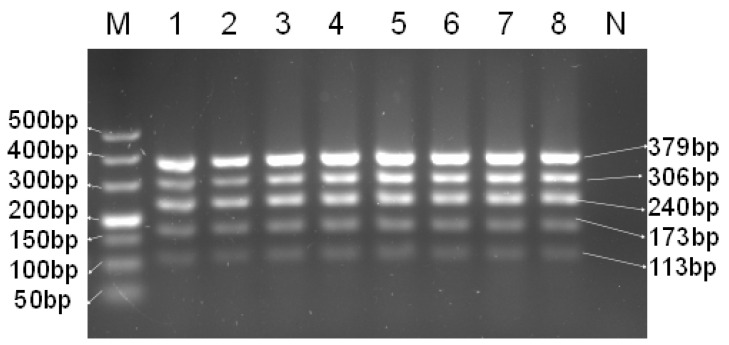
Optimization of annealing temperature of multiplex PCR system. M—DNA ladder (50 bp); 1—60 °C; 2—59.7 °C; 3—59.2 °C; 4—58.5 °C; 5—57.5 °C; 6—56.8 °C; 7—56.3 °C; 8—56 °C; N—blank control.

The results of the gradient optimization of the annealing temperature (Figure 1) demonstrated that all target amplification bands appeared within the range of 56–60 °C. The multiplex PCR system reaction conditions were as follows: 94 °C for 5 min, for 1 cycle; 94 °C for 30 s, 56.8 °C for 30 s, 72 °C for 1 min, for 30 cycles; and 72 °C for 5 min, for 1 cycle. Based on the comprehensive evaluation of fragment amplification brightness, clarity, singularity, the absence of trailing, the lack of non-specific amplification phenomenon, and other aspects, 56.8 °C was selected for the multiplex PCR system.

### 3.2. Specificity of Multiplex PCR

**Figure 2:**
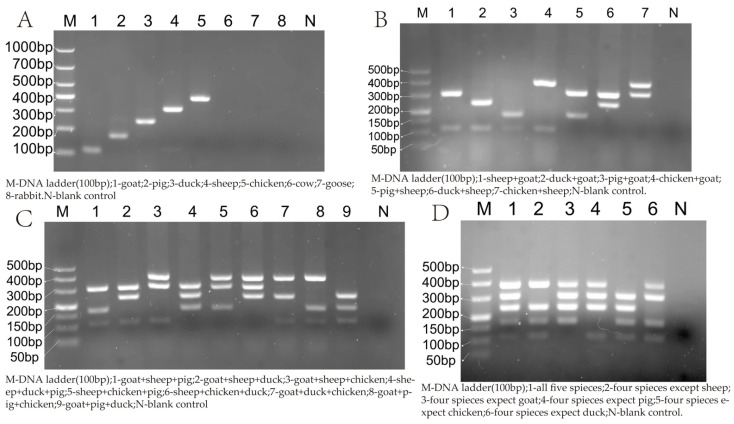
Multiplex PCR system specificity detection. (**A**) Single species detection; (**B**) dual species combination; (**C**) triple species combination; (**D**) quintuple + quadruple species combination.

The DNA of the five target species was both subjected to single and multiplex PCR amplification detected using 2% agarose gel electrophoresis and observed under a gel imaging system. The results are shown in Figure 2, with the amplification products from sheep, goat, pork, chicken, duck, and their various combinations yielding the expected results, with no non-specific amplification, primer competition, and cross-reactivity observed in non-targeted meat (beef, goose and rabbit) and the blank control. The longer amplified fragments (duck: 240 bp, sheep: 306 bp, chicken: 379 bp) exhibit brighter bands than the shorter fragments (goat: 113 bp, pig: 173 bp), which is consistent with the principle that longer DNA fragments intercalate proportionally more fluorescent dye molecules and thus produce a higher band intensity at equivalent molar concentrations [27].

### 3.3. Reproducibility of Multiplex PCR

**Figure 3:**
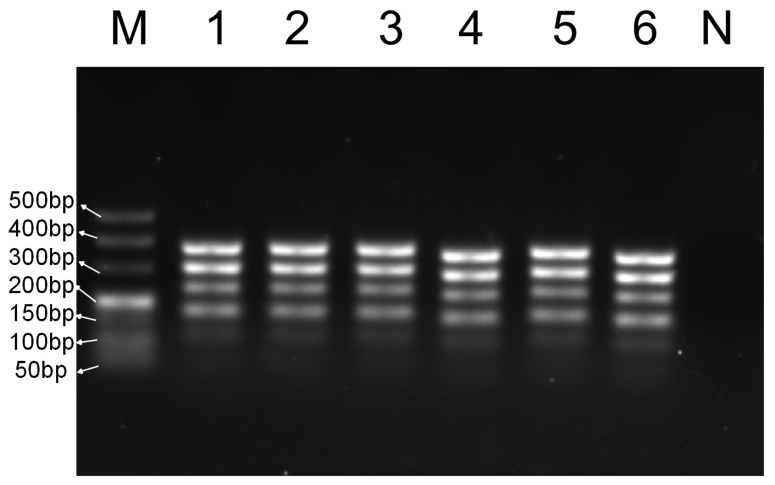
Repeatability test of multiplex PCR system. M—DNA ladder (50 bp); 1~6—6 groups of five target species DNA mixtures; N—blank control.

DNA samples from the five target species (six samples from each species) were subjected to multiplex PCR amplification, and the products were analyzed using gel electrophoresis. As shown in Figure 3, the target bands obtained for each species under identical conditions were uniform and consistent with expectations, demonstrating high reproducibility. The bands enabled the accurate and effective determination of meat sample origins.

### 3.4. Sensitivity of Multiplex PCR

**Figure 4:**
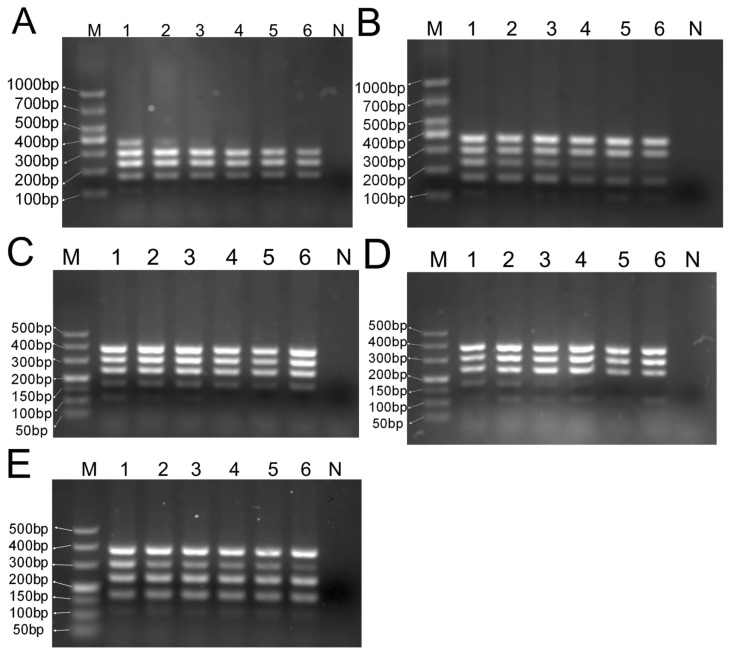
Multiplex PCR system sensitivity assay. (**A**) chicken; (**B**) duck; (**C**) pig; (**D**) goat; (**E**) sheep; M—DNA ladder (DL 100 bp, DL 50 bp); 1—4 ng/μL; 2—2 ng/μL; 3—1 ng/μL; 4—0.5 ng/μL; 5—0.25 ng/μL; 6—0.125 ng/μL; N—blank control.

Five DNA templates were subjected to multiplex PCR amplification and gel electrophoresis detection using concentration gradients ranging from 4 ng to 0.125 ng/μL. As shown in Figure 4, although band intensity gradually decreased with DNA concentration, amplification products from all five experimental animals could be observed at 2 ng/μL. At 1 ng/μL, products from four species could be detected, but not for chicken. At 0.5 ng/μL, sheep, duck, and pig products could still be detected. At the lowest concentration of 0.125 ng/μL, sheep and duck DNA products remained detectable.

### 3.5. Market Adulteration Simulation Experiment

#### 3.5.1. Simulation of Duck Meat Adulteration of Sheep and Goat Meat, and Sheep Meat Adulteration of Goat Meat

**Figure 5:**
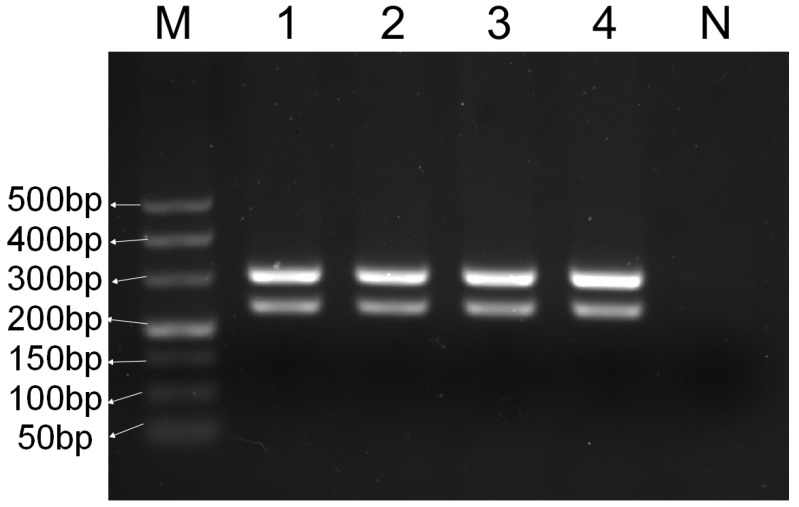
Simulated sheep meat mixture experimental test (duck blended with sheep). M—DNA ladder (50 bp); 1—50% sheep blended with 50% duck; 2—25% sheep blended with 75% duck; 3—10% sheep blended with 90% duck; 4—5% sheep blended with 95% duck; N—blank control.

**Figure 6:**
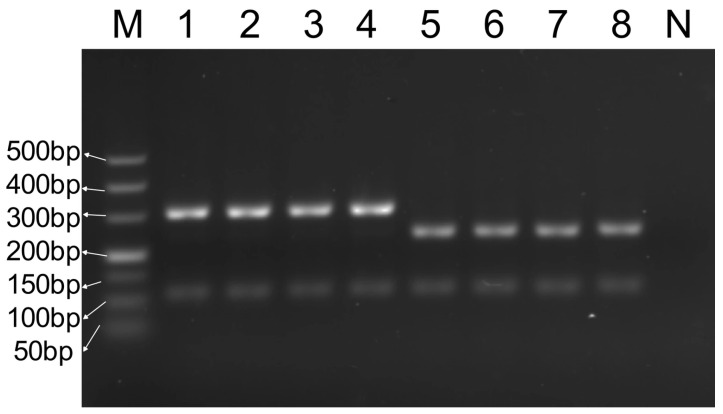
Simulated experimental test charts for goat meat mixes (duck blended with goat, sheep blended with goat). M—DNA ladder (50 bp); 1—50% sheep blended with 50% goat; 2—25% sheep blended with 75% goat; 3—10% sheep blended with 90% goat; 4—5% sheep blended with 95% goat; 5—50% duck blended with 50% goat; 6—25% duck blended with 75% goat; 7—10% duck blended with 90% goat; 8—5% duck blended with 95% goat; N—blank control.

The adulterant meat (duck) was mixed with sheep and goat meat in proportions of 5%, 10%, 25%, and 50%, creating simulated mixtures that might occur in actual markets. After agarose gel electrophoresis, Figure 5 and Figure 6 show that target meat components could still be detected in mixed samples at a mixing ratio of 5%, equivalent to a minimum of 11 mg of non-sheep or non-goat meat.

#### 3.5.2. Analysis of Heat-Treated Meat Product Adulteration Result

**Figure 7:**
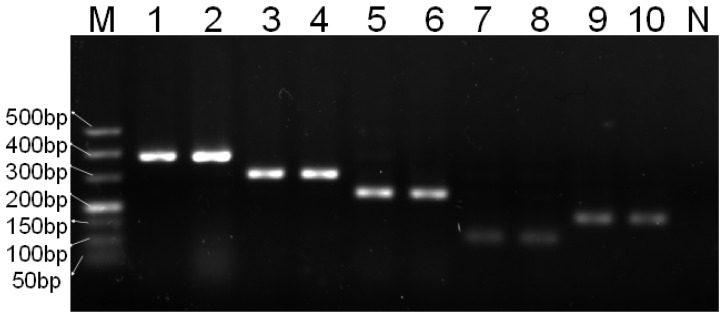
Results of simulated heat-treated meat experiments. M—DNA ladder (50 bp); 1—chicken (fresh); 2—chicken (heat-treated); 3—sheep (fresh); 4—sheep (heat-treated); 5—duck (fresh); 6—duck (heat-treated); 7—goat (fresh); 8—goat (heat-treated); 9—pig (fresh); 10—pig (heat-treated); N—blank control.

As shown in Figure 7, the DNA from the cooked test meat samples showed almost no difference compared with fresh test meat samples, with target bands remaining clear and bright. Although the goat and pig samples possess weaker bands with respect to the other species, they remained detectable in simulation experiments under conditions of 5% concentration or 100 °C. This indicates that the multiplex PCR detection method applies to both fresh meat and heat-processed meat products, making it more adaptable to the evolving market and better aligned with consumer needs.

## 4. Discussion

Food adulteration is a serious problem in many countries, particularly in the meat industry, and has emerged as a global issue. The U.S. Food and Drug Administration (FDA) has estimated global costs between USD 10–15 billion annually, affecting approximately 1% of the global food industry [28]. The global food authenticity testing market, valued at USD 8.68 billion in 2024, is projected to reach USD 16.78 billion by 2033, reflecting the growing demand for authentication technologies [29]. In China, a comprehensive meat food fraud risk database documented 1987 incidents between 2012 and 2021 based on official circular information and media reports, highlighting the prevalence of meat adulteration issues in the world’s largest meat consumer market [30]. Adulteration primarily involves substituting meat products with cheaper ingredients to gain illegal higher profits. This can negatively impact consumers’ health and the economy, and in some cases, violate consumers’ religious laws [31]. This persistent threat underscores the critical need for reliable, cost-effective authentication methods that can be deployed across complex global supply chains.

Various PCR techniques have been used to identify meat, including DNA hybridization, polymerase chain reaction (PCR), DNA sequencing, and polymerase chain reaction-restriction fragment length polymorphism (PCR-RFLP) [32,33,34,35,36]. However, when compared with other techniques such as single nucleotide polymorphism (SNP), DNA barcoding, and polymerase chain reaction random amplified polymorphic DNA (PCR-RAPD), species-specific PCR is considered the best, which can accurately identify species without requiring the restricted digestion or sequencing of PCR products [37]. Furthermore, the combination of species-specific primers in multiplex PCR is the preferred method for the more precise simultaneous and specific identification of different species in a single reaction, reducing both the time and cost [31].

This study utilized the principles of DNA polymerase chain reaction to establish a multiplex PCR detection system for sheep and goat meat adulteration [38,39,40]. Five species-specific primers targeting the mtDNA of the target species were designed [41,42]. The results demonstrated that this system could simultaneously identify five commonly eaten meat types: sheep, goat, pig, chicken, and duck. The system demonstrated significant advantages in sensitivity, specificity, reproducibility, and applicability, providing scientific evidence for authenticating sheep and goat meat in the marketplace.

Multiple techniques for meat species identification using volatile organic compounds, lipids, DNA, and protein molecules are available [43,44]. As proteins are prone to denaturation and degradation during heat treatment, protein-based techniques may no longer be capable of detecting them [45]. DNA-based techniques are fast, reliable, and robust because DNA is a relatively inert molecule, and nucleotide sequence variations enable the tests to distinguish between species [46]. Due to its more stable molecular structure, DNA is less prone to structural alterations during thermal processing and can consistently be detected effectively. Consequently, PCR products of this study obtained for each species were highly consistent and matched expected results when the DNA template concentration, primer concentration, PCR procedures, system conditions, and other aspects remained constant. Additionally, the multiplex PCR system demonstrated high efficiency by identifying five target meat types in a single reaction, significantly improving the detection efficiency while reducing the experimental costs and time. Regarding applicability, in the heat-treated meat simulation experiment, amplification products from heat-treated and fresh meat DNA templates showed that the latter’s bands remained clear and bright, confirming the system’s applicability in thermally processed foods.

DNA-based assay faces the issue of DNA degradation after heating; Galal-Khallaf’s study found that DNA below 600 bp is little affected by temperature, so we designed the specific primers to generate products below 700 bp to ensure the successful establishment of the multiplex PCR system [24]. Studies have been conducted on mitochondrial and chromosomal markers for species identification [43]. However, mitochondrial DNA has a high copy number per cell, enabling the analysis of very small initial samples as well as highly degraded samples in the case of cooked products. Some mitochondrial DNA markers such as ATP synthase subunit 6, cytochrome b, 12S rRNA, 16S rRNA, and NADH dehydrogenase have been used for species identification [34,36,37,46,47]. In this study, the mitochondrial genomes of five species were compared using the ClustalW method, and species-specific primers were designed using five species-specific mitochondrial DNA fragments. The results obtained demonstrated that all five species exhibited clear and bright bands at the target bands, with no non-specific bands observed. Furthermore, no bands were detected in the blank control and non-specific species. The lowest detection concentration of the adulterated meat DNA template was 2 ng/μL, and the detection limits of sheep and duck were as low as 0.125 ng/μL, exceeding group II’s lowest detection concentration of 0.2 ng/μL reported by Li et al. [48]. This far exceeded the requirement of the trace detection of adulterated components in actual samples. Even though the adulteration percentage was controlled at 5%, the detection system was still able to effectively recognize non-target amplification products except goat and sheep.

The current multiple PCR system presents several limitations that warrant consideration. Firstly, the current system provides qualitative rather than quantitative results, identifying the presence of adulterant species but not precisely determining the exact proportion of each meat component in mixed samples. Therefore, integration with qPCR or dPCR techniques is required for precise quantification [49]. Secondly, this study focused on five commonly adulterated species in the market; however, other potential adulterants such as beef, rabbit, or horse meat were not included in the detection panel. Expanding the system to cover additional species would enhance its comprehensiveness, though this may increase the primer optimization complexity and potential for cross-reactivity. Thirdly, while our heat treatment experiment (100 °C, microwave processing) demonstrated system robustness, extremely harsh processing conditions, such as retorting at 121 °C, may cause severe DNA fragmentation beyond the detection capacity of our amplicons. The thermal treatment (microwave oven) employed in this system’s validation remains sufficiently applicable to the majority of market scenarios. Further validation with a broader range of commercial processed products (e.g., sausages, meatballs, canned goods) would enhance the reliability of the method in practical applications. Lastly, this study was conducted under controlled laboratory conditions; field validation involving actual market samples with unknown adulteration histories remains necessary to fully assess the system’s diagnostic accuracy and establish its false-positive/false-negative rates in practical settings. Despite these issues, the market is sufficiently equipped with multiplex PCR systems to determine whether adulteration has occurred.

Building upon the foundation established in this study, several avenues for future development merit exploration. First, integration with real-time qPCR technology could transform this qualitative system into a quantitative assay capable of precisely determining the percentage of each meat component in mixed samples, thereby providing more comprehensive evidence for the legal prosecution of adulteration cases [50]. Second, adaptation to dPCR platforms would further enhance sensitivity and enable absolute quantification without standard curves [51]. Third, expanding the detection panel to include additional species (beef, buffalo, rabbit, horse) and incorporating markers for genetically modified organisms (GMOs) would create a comprehensive food safety screening platform. Finally, integration with blockchain technology could enable transparent traceability throughout the meat supply chain, linking PCR authentication data with production records and distribution networks.

## 5. Conclusions

The multiplex PCR detection system developed in this study can simultaneously identify meat sources from five target species, demonstrating an outstanding performance with the following characteristics: 1. High specificity: no cross-reactivity with non-target species or between target species combinations. 2. Excellent sensitivity: a detection limit of 2 ng/μL for all five species, with sheep and duck detectable at 0.125 ng/μL. 3. Practical applicability: the successful detection of 5% adulteration and a stable performance with heat-treated samples. 4. Good reproducibility: consistent results across six biological replicates. It provides important technical support for the authenticity verification and market supervision of sheep meat and goat meat, with broad application prospects in maintaining food safety and protecting consumer rights. The current system provides only qualitative rather than quantitative results, and the detection panel is limited to five adulterant species commonly found in the Chinese market. Furthermore, extremely harsh processing conditions may cause severe DNA fragmentation, exceeding the detection capability of this system’s amplification fragments. Further work will explore 1. integrating qPCR or dPCR for quantitative analysis; 2. expanding detection to cover additional species (beef, buffalo meat, rat meat, rabbit meat, horse meat); 3. exploring applicability in real-world markets (higher temperatures, autoclaving, fermentation processing, commercial meat products).

## Figures and Tables

**Table 1:** Primer information of multiplex PCR system.

Species	Target Gene	Primer Sequence (5′→3′)	Product Length
Chicken	16S rRNA	F: TGCGTCAAAGCTCCCTCATT R: TTCGCACGGTTAGGATACCG	379 bp
Sheep	COX-2	F: TGCTCTTCCATCCTTGCGAAT R: CGACCTGGAATTGCGTCTGT	306 bp
Pig	16S rRNA	F: TCGCACACGCTTACATCAGT R: TTGGTAAACAGGCGGGGTTT	173 bp
Goat	ND6	F: CTCATCCTCGTCACCGCAAA R: GTGTTTGCGTCTGTTCGTCC	113 bp
Duck	ATP6	F: AAAACGGCCACAAATGAGCC R: GGATTAGTGCGGGGATCAGG	240 bp

## Data Availability

The original contributions presented in the study are included in the article, further inquiries can be directed to the corresponding authors.
